# Fabrication of Electrospun *Juglans regia (Juglone)* Loaded Poly(lactic acid) Scaffolds as a Potential Wound Dressing Material

**DOI:** 10.3390/polym14101971

**Published:** 2022-05-12

**Authors:** Eray Altan, Yasin Karacelebi, Elif Saatcioglu, Songul Ulag, Ali Sahin, Burak Aksu, Alexa-Maria Croitoru, Cosmin Iulian Codrea, Denisa Ficai, Oguzhan Gunduz, Anton Ficai

**Affiliations:** 1 Center for Nanotechnology & Biomaterials Application and Research (NBUAM), Department of Metallurgical and Materials Engineering, Faculty of Technology, Marmara University, Istanbul 34722, Turkey; erai.altan@marmara.edu.tr (E.A.); elifsaatcioglu@marun.edu.tr (E.S.); 2 Center for Nanotechnology & Biomaterials Application and Research (NBUAM), Department of Bioengineering, Faculty of Engineering, Marmara University, Istanbul 34722, Turkey; yasinkaracelebi@marun.edu.tr; 3 Center for Nanotechnology & Biomaterials Application and Research (NBUAM), Department of Metallurgical and Materials Engineering, Institute of Pure and Applied Sciences, Marmara University, Istanbul 34722, Turkey; ulagitu1773@gmail.com; 4 Department of Biochemistry, Faculty of Medicine, Marmara University, Istanbul 34722, Turkey; alisahin@marmara.edu.tr; 5 Department of Medical Microbiology, Faculty of Medicine, Marmara University, Istanbul 34722, Turkey; baksu@marmara.edu.tr; 6 Department of Science and Engineering of Oxide Materials and Nanomaterials, Faculty of Applied Chemistry and Materials Science, University Politehnica of Bucharest, 1–7 Gh Polizu Street, 060042 Bucharest, Romania; alexa_maria.croitoru@upb.ro (A.-M.C.); codrea.cosmin@yahoo.com (C.I.C.); 7 National Centre for Micro and Nanomaterials, University Politehnica of Bucharest, Splaiul Independentei 313, 060042 Bucharest, Romania; denisa.ficai@upb.ro; 8 National Centre for Food Safety, University Politehnica of Bucharest, Splaiul Independentei 313, 060042 Bucharest, Romania; 9 “Ilie Murgulescu” Institute of Physical Chemistry, Romanian Academy, 202 Splaiul Independentei, 060021 Bucharest, Romania; 10 Department of Inorganic Chemistry, Physical Chemistry and Electrochemistry, Faculty of Applied Chemistry and Materials Science, University Politehnica of Bucharest, 1–7 Gh Polizu Street, 060042 Bucharest, Romania; 11 Academy of Romanian Scientists, Ilfov St. 3, 50044 Bucharest, Romania

**Keywords:** electrospinning, polylactic acid, juglone, skin tissue engineering, wound dressing

## Abstract

Juglone (5-hydroxy-1,4-naphthoquinone) (J) is a naphthoquinone structured allelochemical that is mostly found in the roots, leaves, nut-hulls, bark, and wood of walnut (*Juglans regia*). In this study, the biocompatibility, mechanical, thermal, chemical, morphological, and antimicrobial properties of the poly(lactic acid) (PLA) (*w*/*v*)/J (10, 20, 30 mg) electrospun scaffolds were investigated. Based on the results of the study, it was shown that juglone addition increased the antimicrobial properties of the scaffolds against the *Staphylococcus aureus (S. aureus)* and *Escherichia coli (E. coli)*, compared to the neat PLA film after 24 h of contact time. According to the tensile test results, the addition of J made the scaffolds more flexible but decreased the mechanical strength. The cytotoxicity properties of the J-added scaffolds demonstrated a toxic behavior on the first day of incubation. However, with an increase in the J ratio, the fibroblast cell metabolic activity increased for all incubation periods.

## 1. Introduction

Skin is the largest organ in the body, which acts as a barrier against environmental hazards, such as ultraviolet (UV) radiation, chemical, and physical influences, and microorganisms. Skin also prevents dehydration, regulates temperature, and has self-healing properties [1]. Skin disease is one of the most common human illnesses and affects between 30% and 70% of people, regardless of age or ethnicity. Therefore, it directly affects the life quality of individuals [2]. Skin wounds can be treated in many ways, such as by the means of topical medications [3], local steroids [4], oral antibiotics [5], laser therapy [6], phototherapy [7], radiofrequency [8], and skin tissue engineering. The objective of skin tissue engineering is the regeneration of the normal anatomy and physiology of native skin. Full skin regeneration, by means of tissue engineering, requires innovative materials that can restore the epidermal barrier function and dermal properties, as well as the mechanical stability and elasticity [9]. Electrospinning is a unique approach using electrostatic forces to produce fine and continuous fibers. Fiber production, using electrostatic forces, has gained significant attention because of its potential to form fine fibers. Electrospun fibers have a small pore size and high surface area. There is also evidence of sizable static charges in electrospun fibers that could be effectively handled to produce bi- and three-dimensional structures [10]. Recently, there is a growing interest in applying the electrospinning technique for the production of nanofibers that contains active pharmaceutical ingredients, such as plant compounds, due to their enhanced antibacterial, anti-inflammatory, and anti-oxidant properties. Extracts are obtained from either fresh or dried plants by extracting active ingredients from plants using solvents. These solvents can be water or organic solvents [11]. Plants, such as centella asiatica, green tea, mangosteen, tecomella undulata, aloe vera, chamomile, grape seed, calendula officinalis, juglone, indigofera aspalathoides, scrophularia striata, and memecylonedule are some of the used items for wound healing in the field of electrospinning. For instance, Suganya et al. investigated the combined effect of *Cissus quadrangularis* L. (CQ) and hydroxyapatite (HA) by producing PCL-CQ-HA electrospun scaffolds. Enhanced adhesion and proliferation of human fetal osteoblasts (hFOBs) on the composite scaffolds were observed [12]. Moreover, PCL nanofibers with osteo-inductive potential were produced also using Asian Panax Ginseng root extract by Pajoumshariati et al., and the ginseng extract had a positive effect on the hydrophylicity and mechanical properties of the nanofibers [13].

PLA is one of the important upcoming biomaterials due to its advantages, such as biocompatibility and excellent processability, especially by electrospinning. It has high thermo-mechanical properties and displays high biodegradability and bioabsorbability. Moreover, PLA is non-toxic to the human body [14]. Juglone is a major chemical component present in almost all parts of the walnut tree. It is also reported to have anticancer activity against melanoma cell lines [15] and wound healing potential [16]. It has been proven that juglone treatment of skin wounds resulted in a faster rate of growth, migration, and recovered cell morphology [17]. Juglone has been blended with other polymers and different types of nanofibers were electrospun in the literature. Mamipour et al. produced PCL-juglone nanofibers to observe the antibacterial properties of juglone. Antibacterial tests indicated that the prepared nanofibers have a bactericidal effect. The PCL-juglone nanofibers produced a reduction against *Escherichia coli*, *Salmonella enteritidis*, *Staphylococcus aureus*, and *Streptococcus agalactiae* [18]. In another research, it was extracted from the walnut green husk and electrospinning with polyvinyl pyrrolidone (PVP). It exhibited the best inhibitory effect on the proliferation of *Escherichia coli.* Moreover, the antibacterial application potential of the PVP-juglone nanofibers in clinical use is predictable [19]. In this research, PLA (*w*/*v*)/J electrospun scaffolds were designed and studied for their antimicrobial activity against *S. aureus* and *E. coli*. Moreover, the morphological, mechanical, drug release, and in vitro cellular behavior of the electrospun scaffolds have been investigated in order to prove the potential of the scaffolds as wound dressing materials.

## 2. Material and Methods

### 2.1. Materials

PLA 2003D was supplied from Nature Works LLC, USA. J 97% was bought from Sigma-Aldrich, Germany. Dimethylformamide (DMF) ≥ 99% was supplied by Merck, Germany. Dichloromethane (DCM) was obtained from ISOLAB, Germany and Tween 80 was bought from Sigma-Aldrich, Germany.

### 2.2. Preparation of the Electrospinning Solutions

Firstly, PLA (*w*/*v*) was dissolved in 20 mL of DCM: DMF (1:9) and stirred for 2 h using a magnetic stirrer (Wise Stir^®^, MSH-20 A, Wertheim, Germany). After complete mixing, to decrease the surface tension, 3% Tween 80 viscous liquid was added to the PLA (*w*/*v*) solution and the solution was mixed for 15 min. Afterward, different amounts of J (10, 20, and 30 mg), were added to the PLA (*w*/*v*) solution, and further mixed for one hour. After the preparation of the solutions, they were let to dry for around an hour at room temperature before the electrospinning.

### 2.3. Fabrication of the Scaffolds with the Electrospinning Method

**Figure 1:**
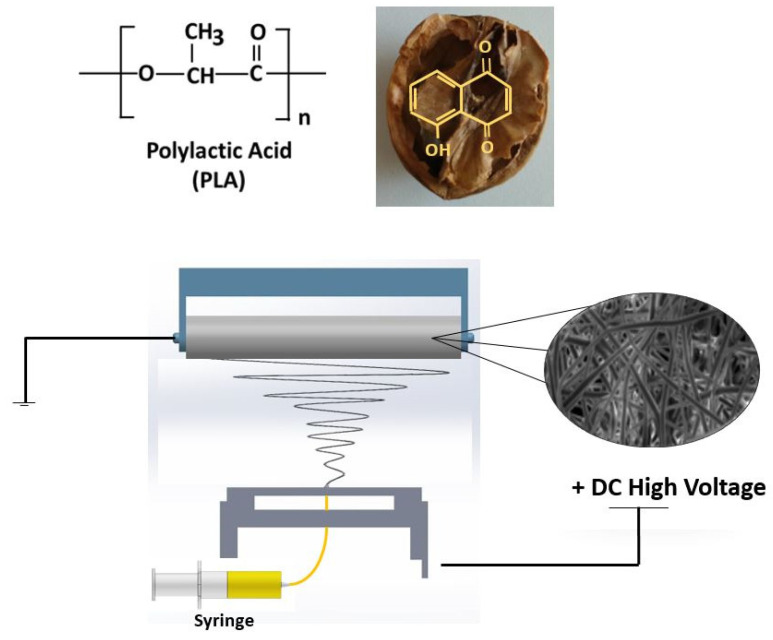
The schematic illustration of the setup.

The experimental setup consists of a high-voltage power supply connected to the needles, a syringe pump (NE-300, New Era Pump Systems, Inc., Farmingdale, NY, USA), a single brass needle (diameter 1.63 mm), and a laboratory-scale electrospinning device (NS24, Inovenso Co., Istanbul, Turkey). An aluminum cylinder covered with greaseproof paper was used to collect the fibers. Plastic syringes filled with 20 mL of solution were used. The collector and the tip of the needle were connected to a high-voltage power supply. Electrospinning parameters were optimized during the electrospinning process and were determined as: 24–26.5 kV operating voltage range, the distance between the needle and the collector was fixed at 12 cm, and the flow rate values varied between 0.3 mL/h and 1 mL/h, The speed of rotation of the collector was fixed to the value of 50 rpm. The electrospinning process was performed at room temperature. The schematic illustration of the electrospinning setup is given in Figure 1. The produced nanofibers were left in the suction head at room temperature for 24 h to remove any remaining solvent.

### 2.4. Characterization of the Scaffolds

The chemical properties of the scaffolds were examined by Fourier transform infrared spectroscopy (FT-IR-4000, JASCO, Easton, MD, USA) with a scanning rate between 4000 and 400 cm^−1^. The small pieces of fibers were placed directly onto the diamond tip of the device. The spectra were obtained by co-adding 32 scans, while the spectral resolution was set at 4 cm^−1^.

The surface morphologies of the scaffolds, as well as the size of the fibers, were observed with a scanning electron microscope (SEM, MA-EVO10, ZEISS). Before the analysis, the scaffolds were coated with gold under vacuum for 60 s using a Quorum SC7620 Mini Sputter Coater. The mean fiber diameter and their distribution were measured by using image software (Olympus AnalySIS designed by Olympus Soft Imaging Solutions (OSIS), Waltham, MA, USA).

The mechanical properties of the scaffolds were determined with uniaxial tensile testing equipment (EZ-LX, Shimadzu, Japan) using a 5 kN load cell. An ASTM D3822 standard was used to perform this test. The force and test speed values were adjusted to 0.1 N and 5 mm/min, respectively. The scaffolds were placed directly between the jaws and analyzed in triplicate, for all concentrations.

The thermal properties of the scaffolds were examined with a differential scanning calorimeter (DSC-6O Plus, Shimadzu, Japan). The heating rate was adjusted at 10 °C/min and the temperature range was between 25 °C and 300 °C.

### 2.5. In Vitro Drug Release Studies

The in vitro release profiles of the J from PLA (*w*/*v*) fibers were carried out in phosphate-buffered saline (PBS) solution (pH 7.4) at 37 °C. For the linear calibration curve of the J, five different drug concentrations were used and the absorbance values were detected at 230–300 nm. To start the drug release test, 5 mg of J-loaded scaffolds were weighted and added into Eppendorf tubes with 1 mL of PBS (pH 7.4). The measurements were taken after 15, 45 min, 1, 2, 3, 4, 6, and 30 h. The fresh PBS solution was used during the test and the absorbance values were detected at 253 nm. The molar extinction coefficient of juglone is ε = 3811 M^−1^ cm^−1^.

The cumulative release percentage of the J was calculated using Equation (1) obtained from the absorbance graph. In this equation, y represents the absorbance values obtained from the drug release at 253 nm and x represents the amount of loaded drug. For the drug release, the amount of drug released in the hour interval was found by dividing the amount of drug released in each time interval by the total drug amount and multiplying by 100. Then, when calculating the cumulative release, the amount of drug released in the previous period was also added to calculate the total amount of drug released during that time period.

y = 0.3512x + 0.11599(1)

### 2.6. Assessment of Antimicrobial Activity

An agar disk diffusion method (ADM) was performed to determine the potential antimicrobial activity properties of the fibers. The *S. aureus* and *E. coli* bacteria were cultivated on Columbia agar (Biomerieux, Lyon, France) medium with 5% sheep blood and left to incubate at 35–37 °C for 24 h. The next day, the colony on the agar was removed and a 0.5 McFarland turbidity cell suspension (1–5 × 10^8^ cfu/mL) was prepared in the Müller–Hinton liquid medium (MHB) (Merck, Germany) in the cell densitometer device (Merck, Germany). The reference strains were diffused on the medium containing 90 mm Müller–Hinton agar (Merck, Germany) with a spreader. The disk samples that were previously sterilized under ultraviolet light were placed on the medium. For *E. coli* ATCC 25922 and *S. aureus* ATCC 29213, the disk containing 10 and 2 µg of ampicillin (AMP) was considered as a control. All samples were also placed in bacteria-free MHB for contamination analysis. Later, the media were incubated at 35–37 °C for 24 h. After incubation, the growth inhibition diameter around the disk was determined in mm.

### 2.7. Biocompatibility Tests

A mouse L929 fibroblast cell line was used in the biocompatibility test to examine cell-material interactions. The L929 cell line was obtained from American type culture collection (ATCC), Manassas, VA, USA. Dulbecco’s Modified Eagle Medium (DMEM) supplemented with PBS, penicillin/streptomycin solution, and L-glutamine was used throughout the experiment. A circular mold of 5 mm diameter and 0.2 mm thickness was used to prepare the samples for the cell culture test. Then, the samples were UV sterilized overnight. Sterilized scaffolds were placed in 96-well plates and fibroblast cells (L929, 5 × 10^3^/well) were plated on the scaffolds. The scaffolds combined with the cells were kept in the incubator at 37 °C and 5% CO_2_ for a week. The MTT (3-(4,5-dimethylthiazol-2-yl)-2,5-diphenyl-2H-tetrazolium bromide) test was performed to observe the metabolic activity of cells and measurements were repeated three times. SEM microscopy was used for cell-material imaging.

The metabolic activity of fibroblast (L929) cells on the scaffolds was quantitatively determined by the MTT test starting on the first day of first culturing and on the fourth and seventh days. After incubation (37 °C, 5% CO_2_), all media inside the wells was discarded, and the scaffolds were rinsed three times with PBS solution. Amounts of 90 μL of fresh medium and 10 μL of MTT solution (5 mg/mL in PBS solution) were added to the freshly washed samples and left in the incubator (37 °C, 5% CO_2_) for 3 h. The medium was discarded and 200 μL of dimethyl sulfoxide (DMSO) was added to the scaffolds to dissolve the formazan crystals and kept in the incubator for 1 h. Finally, the medium was removed from the wells and the absorbance values were measured at 540 nm with a microplate reader.

## 3. Results and Discussions

### 3.1. FT-IR Analysis

**Figure 2:**
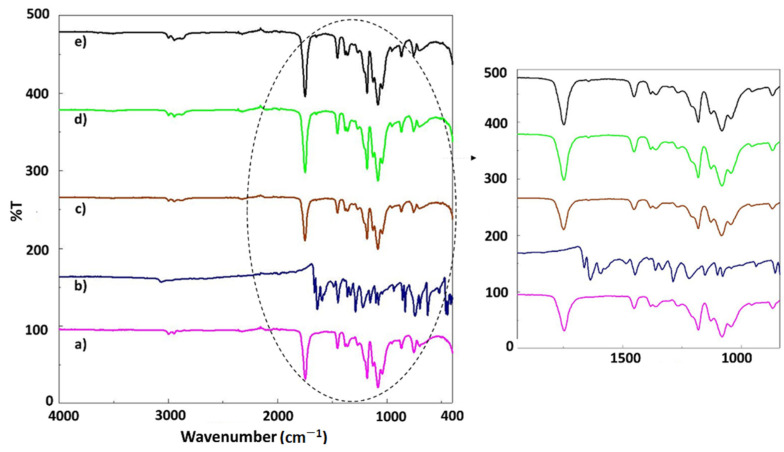
FT-IR spectra of the PLA (*w*/*v*) (**a**), pristine juglone (**b**), and scaffolds: PLA (*w*/*v*)/10 J (**c**), PLA (*w*/*v*)/20 J (**d**), an PLA (*w*/*v*)/30 J (**e**).

In Figure 2a, the main absorption peaks of PLA are shown, such as the peak from 1747 cm^−1^ (C=O vibration), ~1450 cm^−1^ (CH_3_ asymmetrical scissoring), ~1079 cm^−1^ (C–O–C stretching), ~1041 cm^−1^ (C–CH_3_ stretching), and ~865 cm^−1^ (C–COO stretching) [17]. The FT-IR spectrum of the juglone is shown in Figure 2b. The characteristic bands observed at 3060 cm^−1^, 1988 cm^−1^, 1662 cm^−1^, 1590 cm^−1^, 1484 cm^−1^, 1361 cm^−1^, 1288 cm^−1^, 1078 cm^−1^, 833 cm^−1^, 742 cm^−1^, and 518 cm^−1^ are characteristic for pristine juglone. Some C–H stretching and bending weak and strong bands were detected at 3060 cm^−1^ and 1484 cm^−1^. Other peaks observed at 1361 cm^−1^, 1288 cm^−1^, and 1078 cm^−1^ are attributed to the bending and stretching vibrations of the aromatic rings of juglone [20]. The peak observed at 1662 cm^−1^ is corresponded to the stretching vibration of carbonyl groups, which is the principal reversible redox center exhibited in J molecules [21]. The FT-IR spectra of the PLA samples loaded with juglone are similar to that of PLA (*w*/*v*). However, an enhancement in the relative intensity of the peak from 1750 cm^−1^ can be observed due to the increase in the concentration of J. [22]. Since the amount of PLA is high, the samples loaded with J (10–30%) have almost the same FT-IR spectra as the pristine PLA fiber, this being also in good agreement with other studies [10.3390/polym6010093] [23].

### 3.2. Morphology and Surface Properties of the Scaffolds

**Figure 3:**
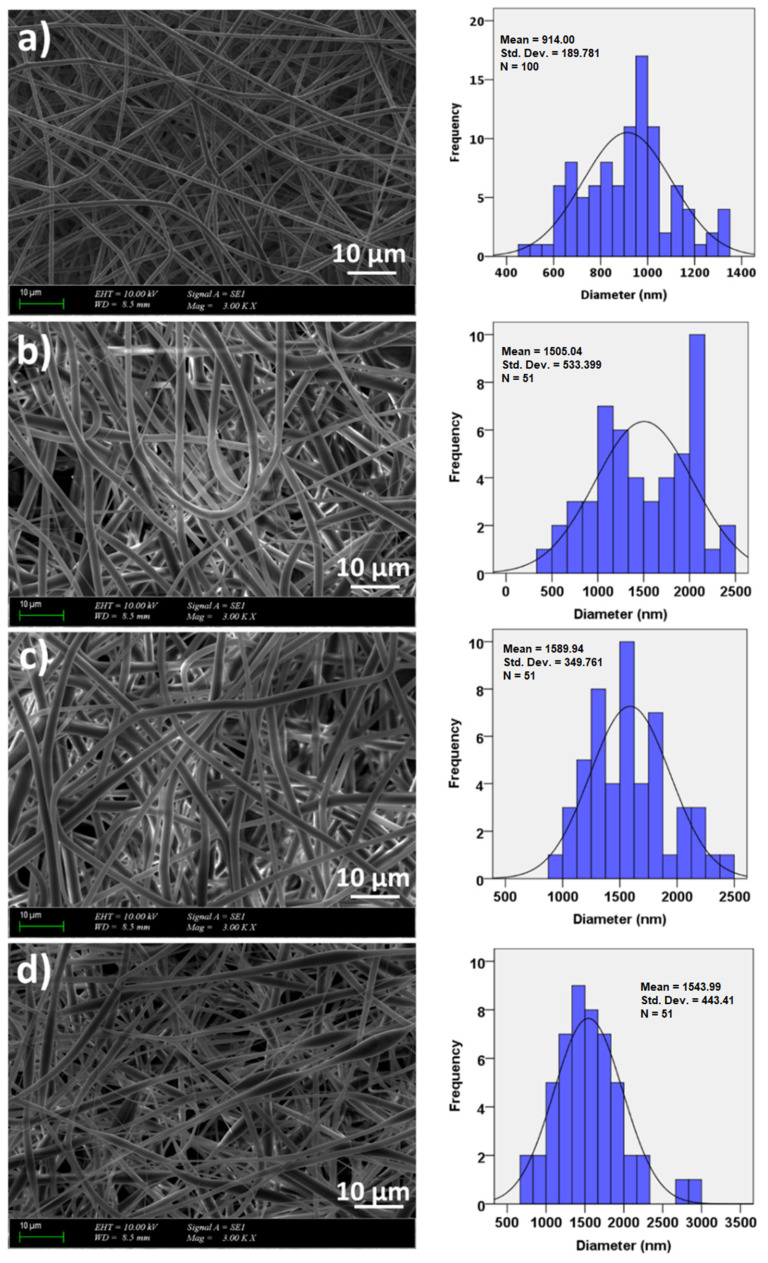
The SEM images of the PLA (*w*/*v*) (**a**), PLA (*w*/*v*)/10 J (**b**), PLA (*w*/*v*)/20 J (**c**), and PLA (*w*/*v*)/30 J (**d**) scaffolds.

The SEM images of the PLA and PLA/J fibers are shown in Figure 3. The uniform and smooth morphologies without any beds were obtained for all groups, except PLA (*w*/*v*)/30 J, and were most probably with a higher concentration of J; this is not uniformly distributed in the whole fiber, and these beaded structures are formed in these heterogeneous areas. Figure 3a shows the SEM image of the PLA (*w*/*v*) fiber with a 914 ± 189 nm histogram graph. By adding 10, 20, and 30 mg of J into the PLA (*w*/*v*) matrix, the diameters of the fibers increased to 1505 ± 535, 1589 ± 349, and 1543 ± 443 nm, respectively (Figure 3b–d). By adding 30 mg of J into the PLA (*w*/*v*) matrix, the diameter of the fibers decreased and it can also be observed that non-uniform and beaded fibers are formed.

### 3.3. Mechanical Properties of the Scaffolds

The stress–strain values of the PLA (*w*/*v*) and J-loaded PLA (*w*/*v*) scaffolds are given in Table 1. The tensile stress value was 3.06 ± 0.32 MPa for the PLA (*w*/*v*) scaffold and the strain value was found to be 4.90 ± 0.63%. With the addition of 10 mg of J into the PLA (*w*/*v*), the tensile stress value decreased to 1.084 ± 0.21 MPa and the tensile strain value increased sharply to the value of 40.72 ± 11.17%. The addition of 20 mg of J into the PLA (*w*/*v*) matrix decreased both the tensile stress (1.045 ± 0.24 MPa) and the strain value (28.16 ± 7.24 Mpa). The highest decrease in tensile stress value was detected with 30 mg of J addition (0.49 ± 0.052 MPa), this fact being justified based on the fusiform morphology of the fibers. The tensile strain value also decreased with 30 mg of J addition (20.67 ± 4.90%), however, it still had a higher value compared to the PLA (*w*/*v*) strain value. These results are in agreement with the literature data [24,25]. It can be concluded that the J addition induced an increase in the tensile strength, making the scaffold more elastic.

### 3.4. Thermal Analysis of the Electrospun Scaffolds

**Figure 4:**
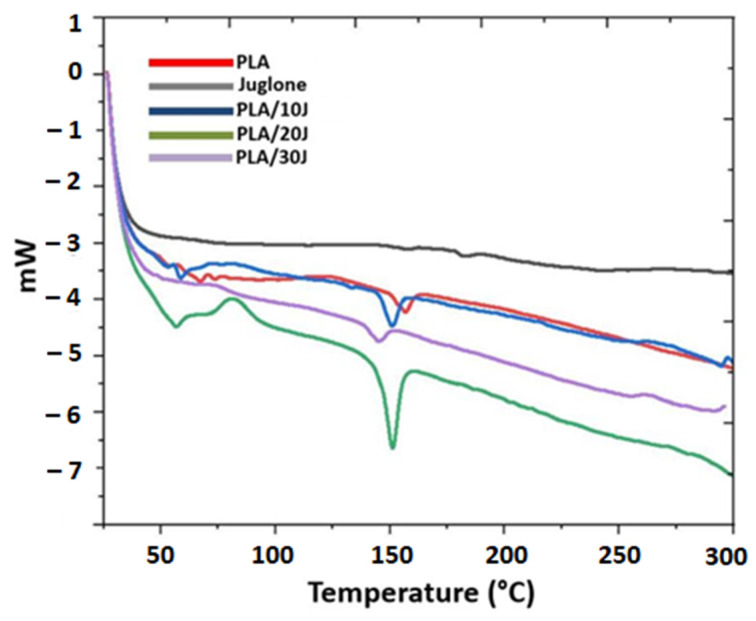
DSC curve of the pristine juglone and PLA/juglone electrospun scaffolds.

Figure 4 shows the DSC curves of the J and J-added PLA (*w*/*v*) scaffolds. PLA can have semi-crystalline or amorphous structures, with a glass transition temperature of about ~55 °C [26]. However, these values may vary according to PLA structures. The T_g_ of PLA was found to be 65.23 °C (−5.57 J/g) in this study, which is close to the T_g_ of standard PLA (55 °C) [27]. The glass transition temperature of the PLA (*w*/*v*) was shifted to 58.23 °C (−10.23 J/g) and 56.92 °C (−15.08 J/g) with 10 mg and 20 mg of J addition, respectively. On the other hand, 30 mg of J addition shifted the T_g_ of PLA to the value of 74.85 °C (4.10 J/g). The melting temperature (T_m_) of PLA is between 130 °C and 180 °C, depending on the L-lactide content and the sort of crystals that originated during crystallization [28]. The melting temperature of the PLA (*w*/*v*) was detected at 157.4 °C (−22.37 J/g) and this value changed with J addition. With the addition of 10 mg of J into the PLA (*w*/*v*), the melting temperature of the PLA (*w*/*v*) decreased to 150.23 °C (−28.55 J/g). The addition of 20 mg of juglone also reduced the melting temperature to 151.35 °C (−40.27 J/g). The highest decrease in the melting temperature was observed in the sample with 30 mg of J added to the PLA (*w*/*v*) fiber scaffold and this value was found to be 144.75 °C (−38.90 J/g).

### 3.5. Drug Release Behavior of the Juglone from the Scaffolds

**Figure 5:**
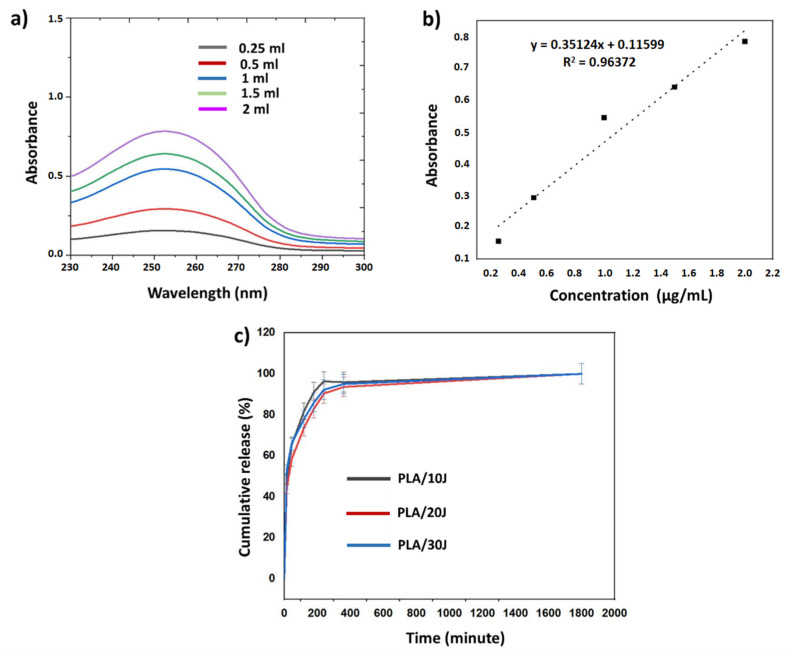
UV spectra of the juglone solution at different concentrations (**a**); calibration curve derived from the spectra (**b**); the cumulative release graph of the three scaffolds (**c**).

The in vitro release of J from the fiber scaffolds was examined in a PBS solution at pH 7.4. The calibration curve of the drug was determined with five different concentrations (from 0.25 to 2 μg/mL) and the graph is represented in Figure 5a. Figure 5b shows the absorbance graph of the J obtained at 253 nm. Figure 5c shows the cumulative release of juglone from different formulations. The results indicated that 48.56, 44.05, and 53.11% of J was released from PLA (*w*/*v*)/10 J, PLA (*w*/*v*)/20 J, and PLA (*w*/*v*)/30 J, respectively, after 15 min of immersion After 4 h, the highest release rate of J (96.29%) corresponds to PLA (*w*/*v*)/10 J. The J release kinetics reached a steady-state after 360 min of immersion. After 1800 min, J was completely released from all the fiber scaffolds. According to the results, it can be said that all formulations have the same release behavior but different release quantity, depending on the amount of J used in the scaffolds. All fiber scaffolds had an initial burst release in the first 15 min and a sustained release after this time point [29]. This behavior is important because a predictable release can be assured. Regardless of the content of the juglone, the cumulative release is quite the same which means that the dose is proportional to the juglone content.

### 3.6. Antimicrobial Properties of the Scaffolds

**Figure 6:**
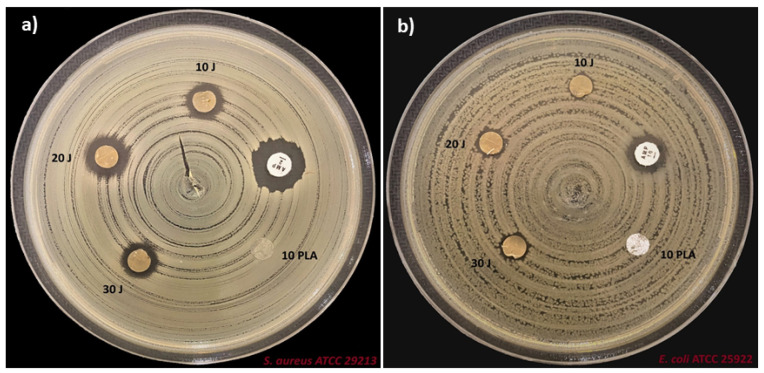
Antimicrobial properties of the scaffolds against the *S. aureus* (**a**) and *E. coli* (**b**).

The antimicrobial efficacy of neat PLA and PLA/J scaffolds against *S. aureus* and *E. coli* is shown in Figure 6. With the addition of J, as a bioactive agent, the antimicrobial properties of the scaffolds were clearly enhanced compared to the neat PLA membrane after 24 h of contact time. As presented in Table 2, PLA scaffolds do not show antibacterial activity against the two bacterial strains. A 100% reduction was considered as the control for the maximum inhibitory effect. According to the presented results, PLA (*w*/*v*)/20 J has the highest bacterial reduction of *S. aureus* (78.5%), compared to a 58% bacterial reduction in the case of *E. coli*. The lowest antibacterial activity of the PLA (*w*/*v*)/10 J was observed against *E. coli* (50%). Therefore, the PLA/J scaffolds exhibited a significant decrease in the growth of the here-studied microorganisms [30]. Comparing these results with that obtained for ampicillin, it is important to mention that, even lower, these alternatives can be suitable in antibiotic-resistant strains where most of the antibiotics have no activity.

In line with this, the antimicrobial activity of the scaffolds is highly dependent on several properties, such as the fiber diameter [31], surface hydrophilicity, roughness [32], stiffness [33], etc.

### 3.7. Cytotoxicity Properties of the Scaffolds

**Figure 7:**
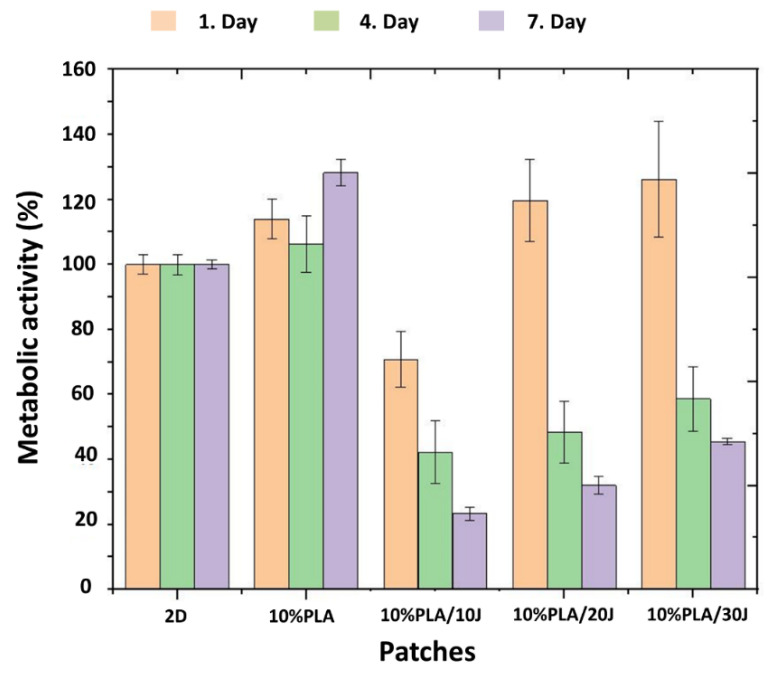
The MTT graph of the 2D (fibroblast cell line) and cells with scaffolds after 1, 4, and 7 days of incubation.

Cell proliferation studies were performed to determine the cytocompatibility of PLA and PLA/J scaffolds, using L929 fibroblast cells. The results are shown in Figure 7. The cells’ metabolic activity, following 1, 4, and 7 days of interaction with the electrospun samples, was calculated as reported to 2D (fibroblast cell line), to which was attributed the value of 100%. All samples showed a cytotoxic behavior on the first day, for the PLA (*w*/*v*)/10 J, with the L929 cells’ metabolic activity being reduced to 70%. For cells cultured with PLA (*w*/*v*)/30 J, cell proliferation was the highest on the first day (125%), meaning that the increase in the J content in the scaffolds leads to an increase in the cell metabolic activity and cell proliferation of the scaffolds. On days 4 and 7 of incubation, cell proliferation was much lower for PLA (*w*/*v*)/10 J, PLA (*w*/*v*)/20 J, and PLA (*w*/*v*)/30 J, revealing a clear cytotoxic behavior, as the metabolic activity decreased with more than 50%, compared to the control. Although PLA (*w*/*v*) samples showed a biocompatible behavior in all 7 days of incubation, with the highest metabolic activity value of 128% (recorded on the seventh day), with the addition of J, the same metabolic activity can be obtained in just one day [34].

The biocompatibility tests performed on the electrospun scaffolds are realized in order to assess the eventual irritant effect of potential leachates in contact applications, such as wound healing scaffolds [35]. In conclusion, the effect of J on cell proliferation depends on the concentration and the duration of cell exposure to the structure.

**Figure 8:**
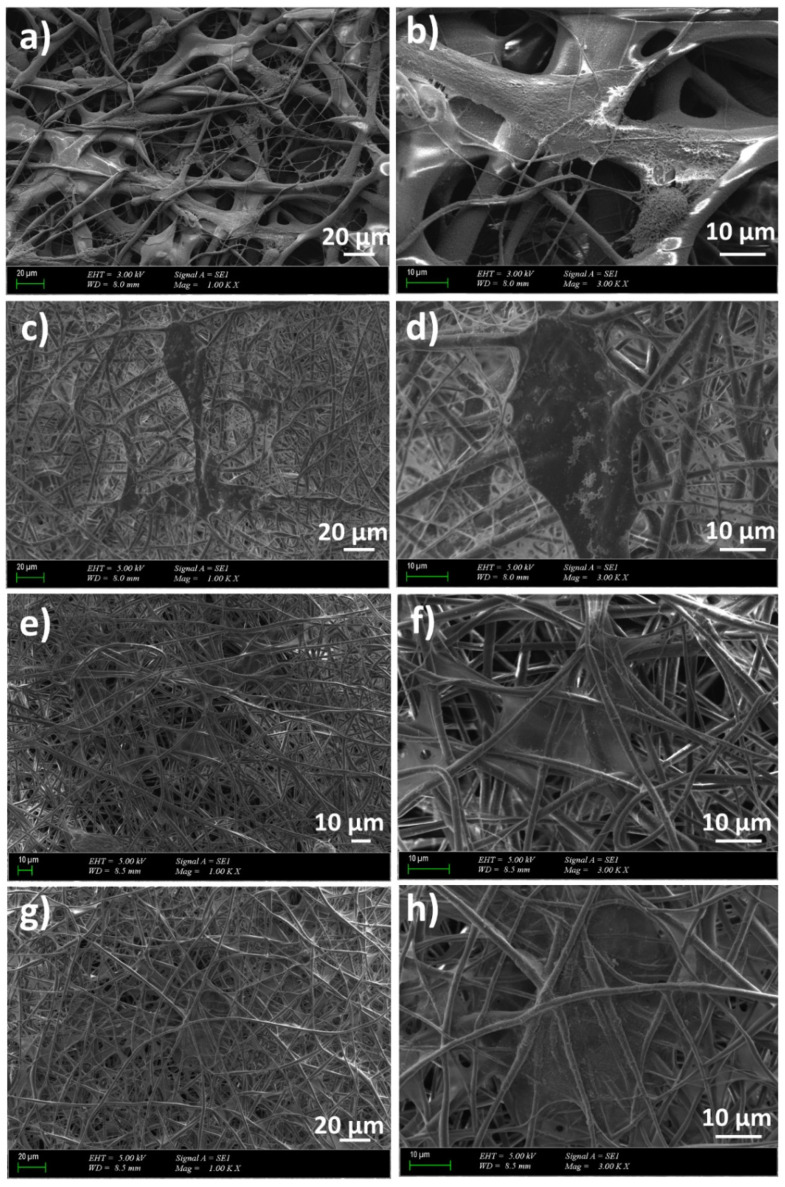
The fibroblast cell morphology on the scaffolds: PLA (*w*/*v*) (**a**,**b**), PLA (*w*/*v*)/10 J (**c**,**d**), PLA (*w*/*v*)/20 J (**e**,**f**), PLA (*w*/*v*)/30 J (**g**,**h**).

Figure 8 shows the fibroblast cell morphology of the nanofiber scaffolds. Because the incubation time was 7 days, the morphology of the fibroblast cells became elongated and was spread on all scaffolds. At higher magnifications, cell proliferation increased with higher J concentrations. These results demonstrated cellular attachment and proliferation of L929 cells on all PLA/J scaffolds, which means that even if the MTT tests highlight cytotoxicity due to the high release of J, the scaffold does not maintain advanced cytotoxicity, and after release, these PLA supports promote cell adhesion and further cell differentiation.

Along with the antimicrobial and even antibiofilm activity, juglone-based formulations can be used in the treatment of melanoma inhibiting angiogenesis at reasonable toxicity [36,37,38]. Moreover, juglone is an efficient antihypertensive agent that acts through multiple vascular mechanisms [39].

## 4. Conclusions

Novel PLA/Juglone scaffolds were successfully prepared using the electrospinning method. The antibacterial activity of PLA/J against *S. aureus* and *E. coli* bacteria demonstrated the potential of the designed scaffolds for the treatment of skin injuries/wounds. Moreover, the release behavior results demonstrated potential for the PLA/J scaffolds to be used as drug delivery systems, having the ability to assure sustained release. The maximum release of the scaffolds was reached in approximately 240 min (96.29% for PLA (*w*/*v*)/10 J). SEM images reported that the J addition increased the diameters of the fiber scaffolds. According to the MTT assay, it can be concluded that juglone-added fiber scaffolds showed a toxic effect compared to the PLA (*w*/*v*). Thus, by reducing the amount of juglone, its toxic effect can be reduced. The results of this study indicate that PLA/J scaffolds are suitable for delivering juglone, a hydrophobic aromatic agent, for antimicrobial purposes; however, their potential antitumoral activity on different dermal tumors should also be evaluated while considering the antitumoral activity of the juglone. 

## Figures and Tables

**Table 1:** Mechanical behaviors of the scaffolds.

Patch	Tensile Strength (MPa)	Strain at Break (%)
PLA (*w*/*v*)	3.06 ± 0.32	4.90 ± 0.63
PLA (*w*/*v*)/10 J	1.084 ± 0.21	40.72 ± 11.17
PLA (*w*/*v*)/20 J	1.045 ± 0.24	28.16 ± 7.24
PLA (*w*/*v*)/30 J	0.49 ± 0.052	20.67 ± 4.90

**Table 2:** Antimicrobial results of the PLA and PLA/J scaffolds.

Scaffolds	*E. coli* ATCC 25922 Zone Diameter (mm)	*S. aureus* ATCC 29213 Zone Diameter (mm)
PLA (*w*/*v*)	0	0
Ampiciline (AMP)	12	14
PLA (*w*/*v*)/10 J	6	10
PLA (*w*/*v*)/20 J	7	11
PLA (*w*/*v*)/30 J	7	9

## Data Availability

The data presented in the article will be available only upon request.
